# The dynamic volatility nexus of geo-political risks, stocks, bond, bitcoin, gold and oil during COVID-19 and Russian-Ukraine war

**DOI:** 10.1371/journal.pone.0286963

**Published:** 2024-02-15

**Authors:** Muneer Shaik, Mustafa Raza Rabbani, Mohd. Atif, Ahmet Faruk Aysan, Mohammad Noor Alam, Umar Nawaz Kayani

**Affiliations:** 1 Mahindra University, School of Management, Hyderabad, Telangana, India; 2 College of Business Administration, University of Khorfakkan, Sharjah, United Arab Emirates; 3 Department of Commerce and Business Studies, Jamia Milia Islamia, New Delhi, India; 4 Hamad Bin Khalifa University, College of Islamic Studies, Qatar Foundation, Doha, Qatar; 5 Department of Accounting, College of Business Administration, University of Bahrain, Sakhir, Bahrain; 6 College of Business, Al Ain University, Abu Dhabi, United Arab Emirates; Queen Mary University of London, UNITED KINGDOM

## Abstract

We investigate the dynamic volatility connectedness of geopolitical risk, stocks, bonds, bitcoin, gold, and oil from January 2018 to April 2022 in this study. We look at connectivity during the Pre-COVID, COVID, and Russian-Ukraine war subsamples. During the COVID-19 and Russian-Ukraine war periods, we find that conventional, Islamic, and sustainable stock indices are net volatility transmitters, whereas gold, US bonds, GPR, oil, and bitcoin are net volatility receivers. During the Russian-Ukraine war, the commodity index (DJCI) shifted from being a net recipient of volatility to a net transmitter of volatility. Furthermore, we discover that bilateral intercorrelations are strong within stock indices (DJWI, DJIM, and DJSI) but weak across all other financial assets. Our study has important implications for policymakers, regulators, investors, and financial market participants who want to improve their existing strategies for avoiding financial losses.

## 1. Introduction

In a short span of time between March 2020 and March 2022, the world has seen two major crises, namely—the covid-19 pandemic and the crisis due to the Russia-Ukraine war. The covid-19 started as a health emergency in China and became pandemic by March 2020. Till July 2022 over 6.4 million died due to the pandemic and over half a billion got infected by the novel coronavirus (Worldometer). During this time, almost the entire world was under strict lockdown leading to disruption in the supply chain, massive loss of employment and a severe decline in stock and commodity prices. While the world was still struggling with the new variants of the novel coronavirus, the geopolitical tensions arising due to the Russia-Ukraine conflict overtook the pandemic as the major threat to economic growth [1]. One thing common to the covid-19 pandemic and the Russia-Ukraine war is disruption of the global supply chain and increased volatility in financial and commodity markets across the globe [2]. Conventional wisdom suggests that to avoid losses investors should diversify across industries and different asset classes [3, 4]. The main purpose of holding different asset classes is risk reduction through diversification [5]. Investors avoid those assets which are highly correlated or go in tandem. However, during the times of crises, even seemingly unrelated assets have strong co-movement, limiting the benefits of diversification [6]. In addition, the decline in stock market indices following terrorist’s attacks and wars suggests that geopolitical risk has important implications for asset prices [7]. Considering this, we investigate the volatility links between various asset classes (equities, commodities, and cryptocurrencies) and geopolitical risk.

The collection of studies highlights a diverse range of financial topics, from the pivotal role of Islamic banks in Saudi Arabia’s monetary policy transmission and the innovative use of social impact Sukuk for migrants, to the nuanced examination of Bitcoin as a potential hedge and safe haven asset, the dynamic interplay of uncertainty across developed economies, and the significant impact of geopolitical conflicts on financial market connectedness [8–12]. Understanding connectedness among various asset classes is of paramount importance for risk management [13, 14]. Although connectedness among various asset classes has always been important, the financial crisis of 2007–08 made this issue even more important. Studies like [15, 16] revealed that volatility spillovers among financial markets across the globe increased substantially during the global financial crisis. The burst in volatility spillover indices during crises is not limited to equity markets alone. There is evidence of increased connectedness among markets for commodities, digital currencies, precious metals, and crude oil. For example, [17], report that connectedness among commodities increased more than threefold after the financial crisis. Recently, the crisis induced by the covid-19 pandemic further strengthened the importance of understanding connectedness across markets and asset classes.

There are many studies which have examined the impact of the covid-19 pandemic on equity markets volatility [6, 17–27]. Every study came to the same conclusion: the volatility increase brought on by the virus’ spread is greater than the volatility brought on by the financial crisis of 2007–2008. utilizing 19 stock market indices’ high-frequency data, [28] show that volatility connectedness increased significantly during the outbreak of the pandemic and remained high till December 2020. The adverse impact of covid-19 was not limited to conventional equity markets alone. The Islamic markets were also badly hit and behaved similar to the conventional markets [29]. For example, [30, 31] report that even the Islamic indices which were safe haven during the financial crisis of 2008 are found to be strongly connected to conventional stock indices and other assets. In addition to the conventional and Islamic equity markets, investment in green assets, bonds, bitcoin, and commodities also exhibited excessive volatility and showed greater connectedness after the pandemic [32–35]. Along with the equity markets worldwide, the corporate bond market was also very badly hit [29, 36]. There was such a severe liquidity crisis that the fed had to intervene [37, 38]. use realized volatility computed from high frequency data to show that correlation between bitcoin, gold, oil, exchange rate and equities increased significantly during the covid-19 pandemic. The causal relationship between geopolitical risk, tourism arrival, and policy uncertainty was examined by Shahzad et al. [39]. The study discovered that geopolitical risk and policy uncertainty have a major impact on tourism arrivals and have serious implications for the expansion of the industry.

While the world is still struggling with covid-19, a new crisis has emerged in the form of Russia-Ukraine war [40]. The geopolitical risk faced by the international markets in general and regional markets in particular increased sharply after Russian invasion of Ukraine [31]. Hence, it becomes imperative to examine the impact of the war between Russia and Ukraine on the connectedness of various asset classes. Few studies such as [41], investigated the impact of foreign sanctions on the firm performance in Russia and concluded that sanctions have a detrimental effect on corporate performance generally, but it is uncertain how they would affect the energy and oligarch-related industries. Evidence of these firms’ readiness for sanctions during the Crimea incident in 2014 is one way that the impact of sanctions may be lessened. Against this backdrop, in this study, we investigate the dynamic volatility connectedness of Geo-Political risk, Stocks, Bond, Bitcoin, Gold and Oil for the period from January 2018 to April 2022. We investigate the connectivity during Pre-COVID, COVID, and Russian-Ukraine war sub sample periods. We find that the conventional, Islamic, and sustainable stock indices are net volatility transmitters during COVID-19 and Russia-Ukraine war periods, whereas Gold, US. Bond, GPR, Oil, and Bitcoin are found to be net volatility recipients throughout the sample periods. We observe that Dow Jones Commodity Index (DJCI) shifted from net recipient of volatility to net transmitter during Russian-Ukraine war period. Further, we find that bilateral intercorrelations are strong within stock indices Dow Jones World Index (DJWI), Dow Jones Islamic Market World Index (DJIM), Dow Jones Sustainability World Index (DJSI), and weak among other financial assets. Our study has beneficial implications for policymakers, regulators, investors, and financial market participants to redevelop their existing strategies to avoid financial losses.

The pandemic and the subsequent Russian invasion of Ukraine is an unprecedented financial event and there is a need to study the investors behavior and asset allocation during extreme situations such as these. Therefore, our study contributes to the growing strands of literature in multiple ways and has several policy implications. First, our topical idea is unique as well as our choice of asset classes geo-political risks, stocks, bond, bitcoin, and gold are uniquely linked and are influenced by these events. Second, the location aspect of our study is general and shows the general view of the investors. This multiplies the implications of our study for the investors, banks, financial institutions, investment firms, allocation, regulators, and central banks of the respective countries. Finally, there are only a few studies using the Time Varying Parameter Vector Auto-regression (TVP-VAR) model, which has several rewards compared to rolling-window based VAR. In doing so, we contribute to the growing body of literature that highlights the impact of short-term crises on the financial market and how different asset classes are linked in the financial markets.

The rest of the paper is organized as follows: section 2 presents the review of the relevant literature; section 3 provides data description; section 4 describes the methodology employed; section 5 contains empirical results; and finally, section 6 concludes.

## 2. Literature review

In the recent time there have been a growing trend of literature on COVID-19, oil crisis and Russia Ukraine war [29, 39–44]. This section presents a brief review of the relevant literature.

[45–47], are some of the earliest studies on the impact of covid-19 on financial markets. Among various economic and health crises including the great depression, the financial crisis of 2007–08 and various other health emergencies, covid-19 has proved to be the most detrimental to the global financial markets [48]. The way financial and commodity markets across the globe have responded to the covid-19 crisis clearly indicates that the spread of the virus is a source of systematic risk [49].

The advantages of diversity and superior performance compared to traditional assets have made socially and environmentally responsible investment more popular in recent years [50–54].

There are many studies which have investigated the role of cryptocurrencies as diversifiers, hedgers or safe haven assets [see, for example, [21]. Bouri et al. find that bitcoin was a safe haven for energy related commodities till 2013, however, after 2013, this role of bitcoin could not be maintained. [55] use hourly data and report that bitcoin act as a hedge as well as safe haven for some currencies. Conlon & McGee[6] on the other hand, show that bitcoin is not a safe haven during the covid-19 pandemic.

The impact of war induced geopolitical risk on financial markets is not a new phenomenon and dates back to World War II [56]. Numerous theoretical and empirical studies have investigated the linkage between stock prices and political risk. [57, 58] document a negative relationship between geopolitical risk and stock market returns. Similarly, surge in stock return volatility in response to increase in geopolitical risk is supported by many studies [19, 54, 59–62]. [57] use daily data of geopolitical risk index developed by [63] and find that precious metals (particularly gold and silver) act as a safe haven for increases in geopolitical risk. They also report a negative response of stocks and bonds to geopolitical risk. Recently [58] also report that world stock markets reacted negatively to the Russia-Ukraine war. Similarly, [1] use the event study method to show that the Russia-Ukraine war had a significant negative impact on European stock markets. Geopolitical risk has an impact on more than just the stock market and oil prices. Geopolitical risk influences digital currency returns and volatility [64].

[65] show that geopolitical risk has a negative impact on oil volatility. [49] use daily data to investigate the relationship between the number of covid-19 positive cases, oil price, economic and political uncertainty, Dow Jones index and the index of geopolitical risk. Using the wavelet-based methods, the authors find that geopolitical risk and economic and political uncertainty are affected by the covid-19 outbreak. Moreover, the results of wavelet causality reveal that geopolitical risk causes US equities, oil, and economic and political uncertainty. Most recently, [31] utilize TVP-var to examine connectedness among equity markets, bonds, bitcoin, oil, gas, wheat and gold. They report that time-varying connectedness changed owing to the geopolitical tensions arising of Russia-Ukraine war. They further report that volatility shocks are mainly propagated by Russian equities, oil, and bitcoin. In addition, gold is found to be the net receiver of volatility spillovers. [34] investigates the linkages between oil and other financial and commodity markets and report that oil is net transmitter of volatility during the war period.

The importance of a study to understand the spillovers among various asset classes during a crisis stems from a variety of factors. One important reason is that, like the covid-19 pandemic, the geopolitical risk posed by a country’s invasion of another is systematic in nature and thus cannot be diversified [57]. Therefore, it is important to understand how the connectedness of various assets changes in response to such shocks. In this light, the present study is an attempt to examine the connectedness among stock market, commodities, bitcoin, and geopolitical risk.

## 3. Data description

We use daily price series data of eight variables namely: Geo-Political Risk index, Dow Jones World Index, US Bond, Gold, Dow Jones Islamic Market Index, Bitcoin, Dow Jones Commodity Index, and WTI Oil price for the overall period from January 2018 to April 2022. The details of the data variables along with the sources used in this study is provided in **Table 1**. We divide the overall sample into three subsample periods namely: Pre-COVID (January 2018 to December 2019), COVID (January 2020 to December 2021), and Russian-Ukraine war (January 2022 to April 2022) time periods. We first convert the non-stationary price series to return series computed as $\log\left(\frac{P_{t}}{P_{t-1}}\right)$, to make the series stationary.

**Table 1:** Source of variables used in the study.

S.No	Name	Full Form	Used as proxy for	Source
1	GPR	Geo-Political Risk Index	Geo-political risk	https://www.matteoiacoviello.com/gpr.htm
2	DJWI	Dow Jones World Index	Conventional Stocks	www.spglobal.com
3	US. Bond	Market Yield on U.S. Treasury Securities at 10-Year Constant Maturity	Bonds	https://fred.stlouisfed.org/
4	Gold	Gold Spot Prices	Gold	https://www.gold.org/goldhub/data
5	DJIM	Dow Jones Islamic Market Index	Islamic Stocks	www.spglobal.com
6	Bitcoin	Bitcoin Prices	Cryptocurrency	www.spglobal.com
7	DJCI	Dow Jones Commodity Index	Commodities	www.spglobal.com
8	DJSI	Dow Jones Sustainable Index	Sustainable Stocks	www.spglobal.com
9	Oil	West Texas Instrument (WTI) Crude Oil Prices	Oil	https://fred.stlouisfed.org/

**Fig 1:**
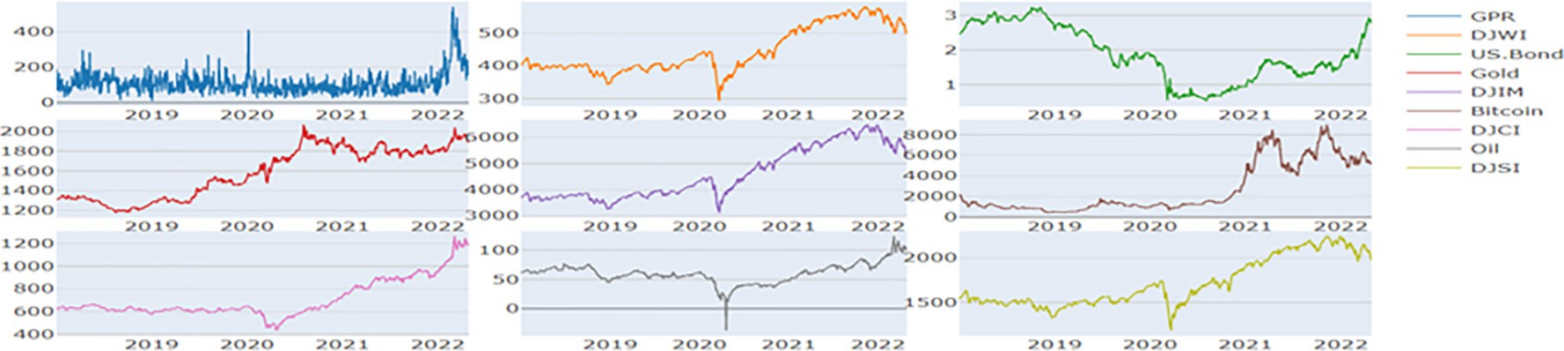
Plots of price series. **Note:** The X-axis denotes the time period from January 2018 to April 2022.

**Fig 2:**
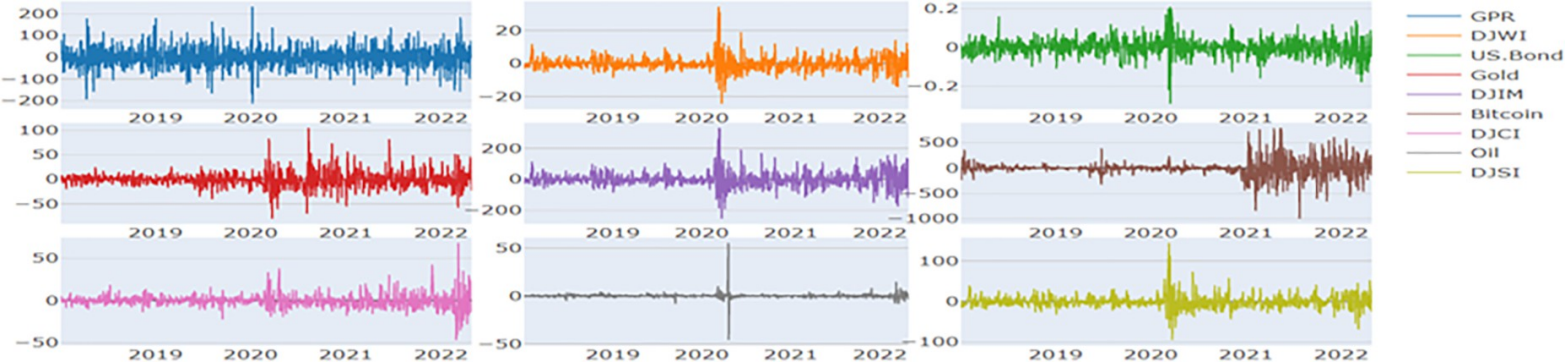
Plots of return series. **Note:** The X-axis denote the period from January 2018 to April 2022.

**Figs 1 and 2** display the plots of the prices and return series respectively. **Table 2** shows the descriptive statistics of the returns for the full sample period from January 2018 to April 2022. During this period, it is observed that the mean return of all the financial assets is negative. We observe Bitcoin to have highest variance (24337.428) and the US.Bond to have the lowest variance (0.002). We find all the assets to be positively skewed except the US.Bond. We observe all the stock indices to have high kurtosis values and are not normally distributed as per the Jarque Bera (JB) test. The return series are observed to be stationary in nature as per ERS unit root test (Stock et.al., 1996). Furthermore, we observe that the return series exhibit ARCH errors based on [66] weighted portmanteau test (Q2(10)). Since we observe ARCH errors, it becomes appropriate to apply multivariate GARCH procedure.

**Table 2:** Summary statistics of the return series (January 2018 to April 2022).

	GPR	DJWI	US bond	Gold	DJIM	Bitcoin	DJCI	Oil	DJSI
**Mean**	0.034	-0.09	0	-0.557	-1.558	-2.878	-0.539	-0.041	-0.412
**Variance**	2540.593	18.67	0.002	245.709	2416.095	24337.428	63.839	7.751	270.299
**Skewness**	0.087 (0.243)	1.094*** (0.000)	-0.068 (0.361)	0.612*** (0.000)	0.741*** (0.000)	0.137* (0.067)	0.717*** (0.000)	3.335*** (0.000)	1.114*** (0.000)
**Ex. Kurtosis**	1.742*** (0.000)	9.479*** (0.000)	3.224*** (0.000)	5.852*** (0.000)	6.205*** (0.000)	7.119*** (0.000)	9.813*** (0.000)	212.855*** (0.000)	10.905*** (0.000)
**JB**	137.326*** (0.000)	4238.893*** (0.000)	466.478*** (0.000)	1600.787*** (0.000)	1822.676*** (0.000)	2273.396*** (0.000)	4405.638*** (0.000)	2031388.007*** (0.000)	5549.222*** (0.000)^.^
**ERS**	-3.801*** (0.000)	-10.524*** (0.000)	-9.830*** (0.000)	-15.732*** (0.000)	-10.054*** (0.000)	-13.824*** (0.000)	-13.516*** (0.000)	-9.444*** (0.000)	10.484*** (0.000)
**Q(10)**	214.653*** (0.000)	40.079*** (0.000)	10.551*** (0.054)	25.310*** (0.000)	32.068*** (0.000)	9.623*** (0.083)	24.052*** (0.000)	81.135*** (0.000)	42.970*** (0.000).
**Q2(10)**	259.525*** (0.000)	596.001*** (0.000)	469.986*** (0.000)	105.936*** (0.000)	611.688*** (0.000)	241.624*** (0.000)	397.962*** (0.000)	234.751^--^- (0.000)	504.419*** (0.000)

**Note:** ***, **, * represents level of significance at 1%, 5%, and 10% respectively. Here, JB denotes Jarque-Bera (1980) normality test, ERS denotes Stock et.al. (1986) unit root test, and Q2(10) denote Fisher and Gallagher (2012) weighted portmanteau test. For skewness, and kurtosis, we use D’Agostino (1970) test, and Anscombe and Glynn (1983) tests respectively.

## 4. Methodology

### 4.1. TVP-VAR based dynamic connectedness approach

[67–70] techniques, which combines the time-varying VAR (TVP-VAR) model with [10] popular model, was used to measure dynamic connectedness between financial assets and uncertainty caused due to infectious diseases. To extract the connectedness indices, we used the TVP-VAR model, which has several advantages over the rolling-window-based VAR. The TVP-VAR model is unaffected by outliers [38, 67, 71, 72] and is similar to that of Antonakakis & Gabauer,[69] who used Bayesian VAR techniques to overcome the constraints of rolling-window-based VAR.

It should be highlighted that the connectedness approach developed by [10, 73] is based on the generalised impulse response functions (GIRFs) proposed by [74, 75]. The benefit of GIRFs is that they are independent of variable ordering and may be understood as the J-step forward impact of a shock in variable i on variable j. Similarly, the volatility impulse response function (VIRF) depicts the influence of a shock in variable i on the conditional volatilities of variable j, which may be represented as:

$$\psi^{g}=VIRF(J,\delta_{j,t},F_{t-1})=E(H_{t+J}|\epsilon_{j,t}=\delta_{j,t},F_{t-1})-E(H_{t+J}|\epsilon_{j,t}=0,F_{t-1})$$ (1)

where *δ*_*j*,*t*_ is a selection vector with a one at the *jth* point and a zero otherwise.

The generalised forecast error variance decomposition (GFEVD) is computed based on the VIRF and may be understood as the variance share one variable explains on others. These variance shares are normalised such that each row amounts to one, indicating that all variables explain 100 percent of variable i’s prediction error variance. This is calculated in the following manner:

$${\tilde{\phi }}_{ij,t}^{g}(J)=\frac{\sum_{t=1}^{J-1}\psi_{ij,t}^{2,g}}{\sum_{j=1}^{N}\sum_{t=1}^{J-1}\psi_{ij,t}^{2,g}}$$ (2)

Where $\sum_{j=1}^{N}{\tilde{\phi }}_{ij,t}^{g}(J)=1$ and $\sum_{i,j=1}^{N}{\tilde{\phi }}_{ij,t}^{g}(J)=N$. The numerator indicates the cumulative effect of the ith shock, whereas the denominator represents the aggregate cumulative effect of all shocks. Using the GFEVD, the total connectedness index (TCI) may be calculated as follows:

$$C_{t}^{g}(J)=\frac{\sum_{i,j=1,i\neq j}^{N}{\tilde{\phi }}_{ij,t}^{g}(J)}{N}$$ (3)

Following that, the spillovers variable i transfers to variables j, which are referred to as total directional connectedness TO others, are computed as follows:

$$C_{i\to j,t}^{g}(J)=\frac{\sum_{j=1,i\neq j}^{N}{\tilde{\phi }}_{ji,t}^{g}(J)}{\sum_{j=1}^{N}{\tilde{\phi }}_{ji,t}^{g}(J)}$$ (4)

In the following phase, the spillovers variable i gets from variables j, known as total directional connectedness FROM others, are determined as follows:

$$C_{i\leftarrow j,t}^{g}(J)=\frac{\sum_{j=1,i\neq j}^{N}{\tilde{\phi }}_{ij,t}^{g}(J)}{\sum_{j=1}^{N}{\tilde{\phi }}_{ij,t}^{g}(J)}$$ (5)

Subtracting the two previously mentioned measures yields the net total directional connectedness, which may be read as the effect variable i has on the examined network:

$$C_{i,t}^{g}(J)=C_{i\to j,t}^{g}(J)-C_{i\leftarrow j,t}^{g}(J)$$ (6)

If variable i’s net total directional connectedness is positive (negative), it signifies that variable i is a net shock transmitter (receiver) or that variable i is driving (being driven by) the network.

Finally, the net pairwise directional connectedness (NPDC) between variables i and j is calculated as:

$${NPDC}_{ij}(J)={\tilde{\phi }}_{ji,t}^{g}(J)-{\tilde{\phi }}_{ij,t}^{g}(J)$$ (7)

where variable i dominates (is dominated by) variable j, as shown by a positive (negative) *NPDC*_*ij*_.

## 5. Empirical results

### 5.1 Average dynamic connectedness

The averaged dynamic connectedness measures are shown in **Table 3**. The dynamic (cTCI) and static (TCI) total connectedness index are observed to be highest among the variables during Russia-Ukraine war period valued at 61.54 and 54.71 respectively. We observe that cTCI (47.81) and TCI (42.50) values to be lowest during Pre-COVID sample period. Furthermore, the statistics clearly indicate that Dow Jones World Index (DJWI) is the net transmittor of volatility to all other variables during the overall sample period, Pre-COVID, COVID, and also during Russia-Ukraine war period. The DJWI is followed by Dow Jones Islamic Market Index (DJIM), Dow Jones Sustainability Index (DJSI), and Dow Jones Commodity Index (DJCI) as the net transmittors of volatility to the rest of the variables under study. However, the major net recepient of volatility from all the other variables is observed to be Gold followed by US. Bond, Geo-Political Risk Index (GPR), Bitcoin, and Oil during full sample, and COVID period. During Pre-COVID period, we observe the U.S. Bond, Oil, GPR, Gold, and Bitcoin to be the net recepients of volatility from all the other variables. Whereas during Russia-Ukraine war period, we find that Gold, Bitcoin, U.S. Bond, GPR and Oil to be the net recepients. Overall the findings suggest that irrespective of the subsample time periods, conventional stocks (DJGI) are observed to be the net transmittors of volatility to all other variables.

**Table 3:** Average dynamic connectedness.

a) FULL (JANUARY 2018 to April 2022											b) Pre-COVID (January 2018 to December 2019										
	**GPR**	**DJWI**	**US bond**	**Gold**	**DJIM**	**Bitcoin**	**DJCI**	**Oil**	**DJSI**	**From**		**GPR**	**DJWI**	**US bond**	**Gold**	**DJIM**	**Bitcoin**	**DJCI**	**Oil**	**DJSI**	**From**
GPR	88.51	1.46	0.59	0.70	1.52	1.75	2.28	1.63	1.57	11.49	GPR	89.97	1.46	0.59	0.44	1.53	1.11	1.16	1.88	1.87	10.03
DJWI	0.27	28.82	4.31	2.56	27.53	1.74	6.12	2.13	26.53	71.18	DJWI	0.35	29.01	4.79	2.71	28.25	0.29	4.91	2.86	26.83	70.99
US bond	0.79	8.88	65.37	2.43	6.57	0.86	4.32	2.47	8.30	34.63	US bond	1.33	9.09	63.94	3.29	7.33	0.64	3.16	2.50	8.71	36.06
Gold	1.27	3.60	4.01	74.22	3.57	1.99	6.20	1.28	3.87	25.78	Gold	1.54	2.24	4.37	79.04	1.74	0.41	6.65	1.83	2.18	20.96
DJIM	0.30	28.43	3.71	2.70	30.32	1.87	5.17	1.85	25.66	69.68	DJIM	0.36	28.88	4.55	2.75	30.29	0.30	4.26	2.80	25.81	69.71
Bitcoin	0.59	4.32	0.71	1.61	4.53	81.16	2.67	0.78	3.63	18.84	Bitcoin	0.18	1.51	0.79	0.35	1.71	93.27	0.38	0.47	1.34	6.73
DJCI	1.01	9.92	2.57	3.36	8.06	2.17	46.75	17.20	8.96	53.25	DJCI	0.57	7.95	2.02	3.10	6.65	0.63	47.83	23.22	8.05	52.17
Oil	0.30	4.33	2.03	0.78	3.43	0.70	19.88	64.64	3.90	35.36	Oil	0.42	5.49	2.11	0.90	5.03	0.60	26.08	54.26	5.11	45.74
DJSI	0.25	27.71	4.07	2.77	26.01	1.53	5.84	1.95	29.85	70.15	DJSI	0.34	27.89	4.53	2.81	26.33	0.28	5.20	2.71	29.91	70.09
To	4.78	88.65	22.01	16.92	81.23	12.61	52.48	29.29	82.41	390.37	To	5.10	84.51	23.74	16.34	78.58	4.27	51.79	38.27	79.89	382.47
Inc. Own	93.28	117.47	87.37	91.13	111.55	93.77	99.23	93.93	112.26	cTCI/TCI	Inc. Own	95.06	113.52	87.68	95.39	108.86	97.54	99.62	92.53	109.81	CTCI/TCI
Net	-6.72	17.47	-12.63	-8.87	11.55	-6.23	-0.77	-6.07	12.26	48.80/43.37	Net	-4.94	13.52	-12.32	-4.61	8.86	-2.46	-0.38	-7.47	9.81	47.81/42.50
NPT	2.00	8.00	1.00	0.00	7.00	3.00	5.00	4.00	6.00		NPT	2.00	7.00	2.00	3.00	6.00	4.00	4.00	3.00	5.00	
C) COVID (JANUARY 2020 to Decenber 2021 d) WAR January 2022 to April 2022																					
	**GPR**	**DJWI**	**US bond**	**Gold**	**DJIM**	**Bitcoin**	**DJCI**	**Oil**	**DJSI**	**From**		**GPR**	**DJWI**	**US bond**	**Gold**	**DJIM**	**Bitcoin**	**DJCI**	**Oil**	**DJSI**	**From**
GPR	85.11	1.75	1.89	0.67	1.49	4.68	1.87	1.22	1.32	14.89	GPR	81.79	4.23	0.93	1.88	5.51	2.02	0.27	0.19	3.18	18.21
DJWI	0.29	27.62	3.59	3.22	25.91	3.25	8.23	1.65	26.25	72.38	DJWI	1.28	27.33	1.49	1.76	26.65	14.38	2.14	1.55	23.43	72.67
US bond	0.68	8.43	66.07	2.35	6.50	1.54	4.18	2.12	8.13	33.93	US bond	2.64	4.33	69.61	1.79	2.82	1.28	7.18	5.45	4.89	30.39
Gold	0.49	5.59	5.73	64.07	6.28	5.02	5.56	1.25	6.01	35.93	Gold	2.15	5.37	5.24	50.87	3.96	0.58	15.85	8.89	7.09	49.13
DJIM	0.32	27.03	2.93	3.43	28.94	3.30	6.74	1.30	26.01	71.06	DJIM	1.56	27.36	0.78	1.14	29.22	15.03	1.44	1.16	22.31	70.78
Bitcoin	0.43	6.78	0.70	4.25	6.79	67.94	5.77	1.40	5.94	32.06	Bitcoin	0.80	18.67	0.98	0.15	19.52	41.76	0.70	2.25	15.17	58.24
DJCI	1.05	11.70	2.53	3.40	9.45	4.81	46.05	10.85	10.17	53.95	DJCI	1.39	3.77	5.30	11.87	2.86	0.69	38.59	30.98	4.54	61.41
Oil	1.13	3.14	1.14	0.41	2.15	2.16	11.75	75.95	2.17	24.05	Oil	1.27	2.74	6.49	7.39	2.28	0.52	33.28	41.99	4.05	58.01
DJSI	0.30	26.56	3.27	3.58	25.07	2.98	7.74	1.65	28.86	71.14	DJSI	0.93	25.56	1.71	3.20	23.70	12.95	3.00	2.48	26.48	73.52
To	4.69	90.97	21.78	21.30	83.63	27.73	51.84	21.46	86.00	409.38	To	12.00	92.03	22.93	29.18	87.29	47.44	63.86	52.96	84.66	492.35
Inc. Own	89.79	118.59	87.85	85.37	112.57	95.66	97.89	97.41	114.87	cTCI/TCI	Inc. Own	93.80	119.35	92.54	80.05	116.52	89.21	102.45	94.95	111.13	cTCI/TCI
Net	-10.21	18.59	-12.15	-14.63	12.57	-4.34	-2.11	-2.59	14.87	51.17/45.49	Net	-6.20	19.35	-7.46	-19.95	16.52	-10.79	2.45	-5.05	11.13	61.54/54.71
NPT	0.00	8.00	2.00	1.00	6.00	4.00	5.00	3.00	7.00		NPT	4.00	8.00	2.00	0.00	7.00	3.00	4.00	2.00	6.00	

Note: Values reported are variance decompositions based on 100-day ahead forecasts. NPT represents Net Pairwise Transmitter.

### 5.2 Dynamic total connectedness

**Fig 3:**
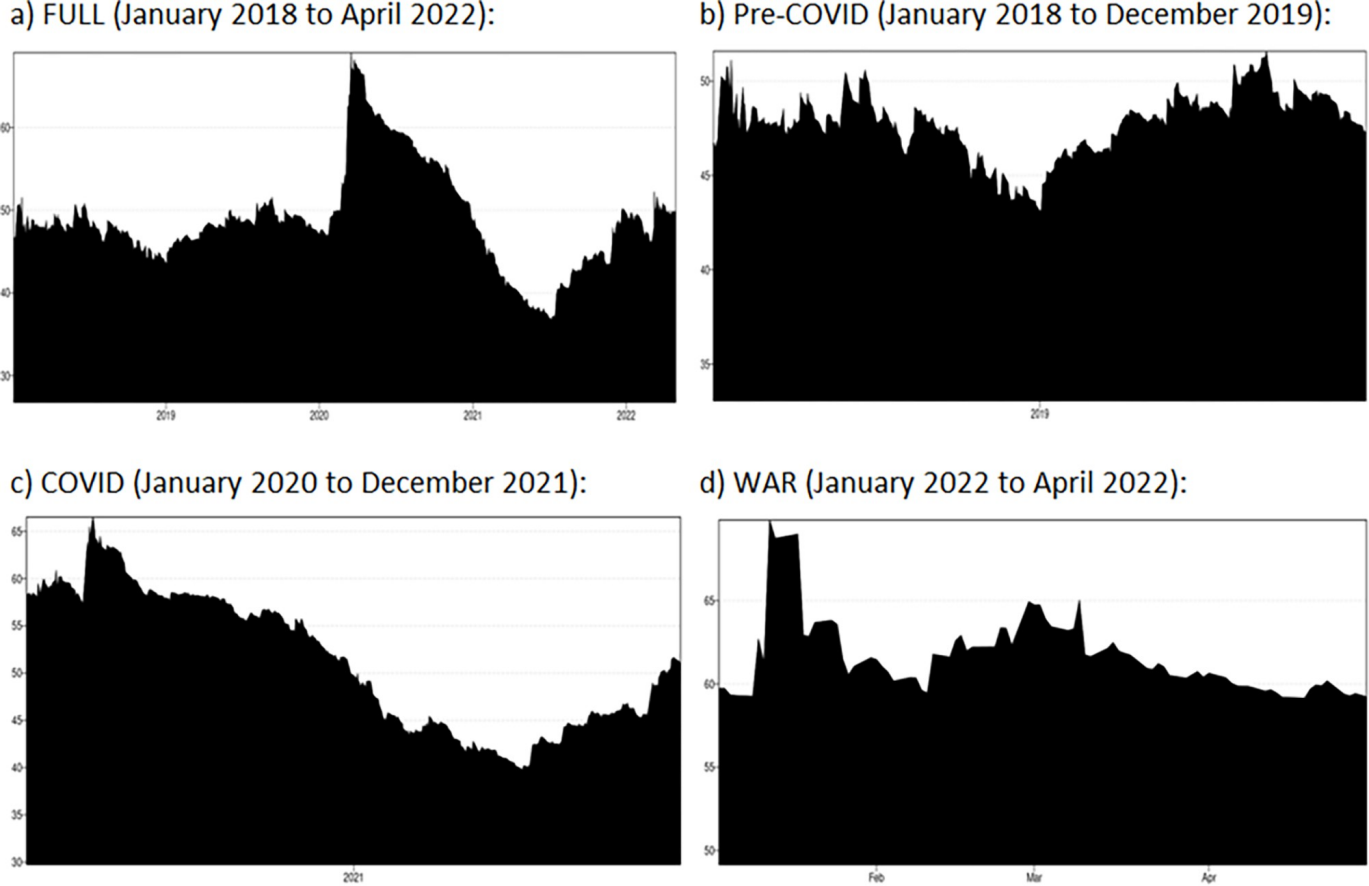
Dynamic total connectedness during different sample periods. a) FULL (January 2018 to April 2022): b) Pre-COVID (January 2018 to December 2019): c) COVID (January 2020 to December 2021): d) WAR (January 2022 to April 2022): Note: The X-axis denotes the time line. The Y-axis denotes the range of TCI index from 0% to 100%.

**Fig 3A** displays the dynamic total connectedness (Gabauer, 2021; Chatziantoniou & Gabauer, 2021) which ranges between 38% (during mid 2021) and 70% (during early 2020) for the overall sample period from January 2018 to April 2022. During Pre-COVID sample period, **Fig 3B** shows that the value ranges between 38% and 52%. During COVID period, **Fig 3C** shows that the dynamic total connectedness ranges between 38% and 70%; whereas during the Russian-Ukraine war period, **Fig 3D** shows that the value ranges between 58% and 70%. This practically implies that connectedness across the financial assets is strong and time-varying, a fact that is often obscured by the static nature of the TCI. To be more specific, two major spikes can be seen in Fig 3, the first of which may be linked to the pandemic COVID-19 which disrupted the global financial markets during the early 2020; and the second is during the escalation of tensions due to Russian-Ukraine war during the early 2022.

### 5.3 Net Directional connectedness measures

**Fig 4:**
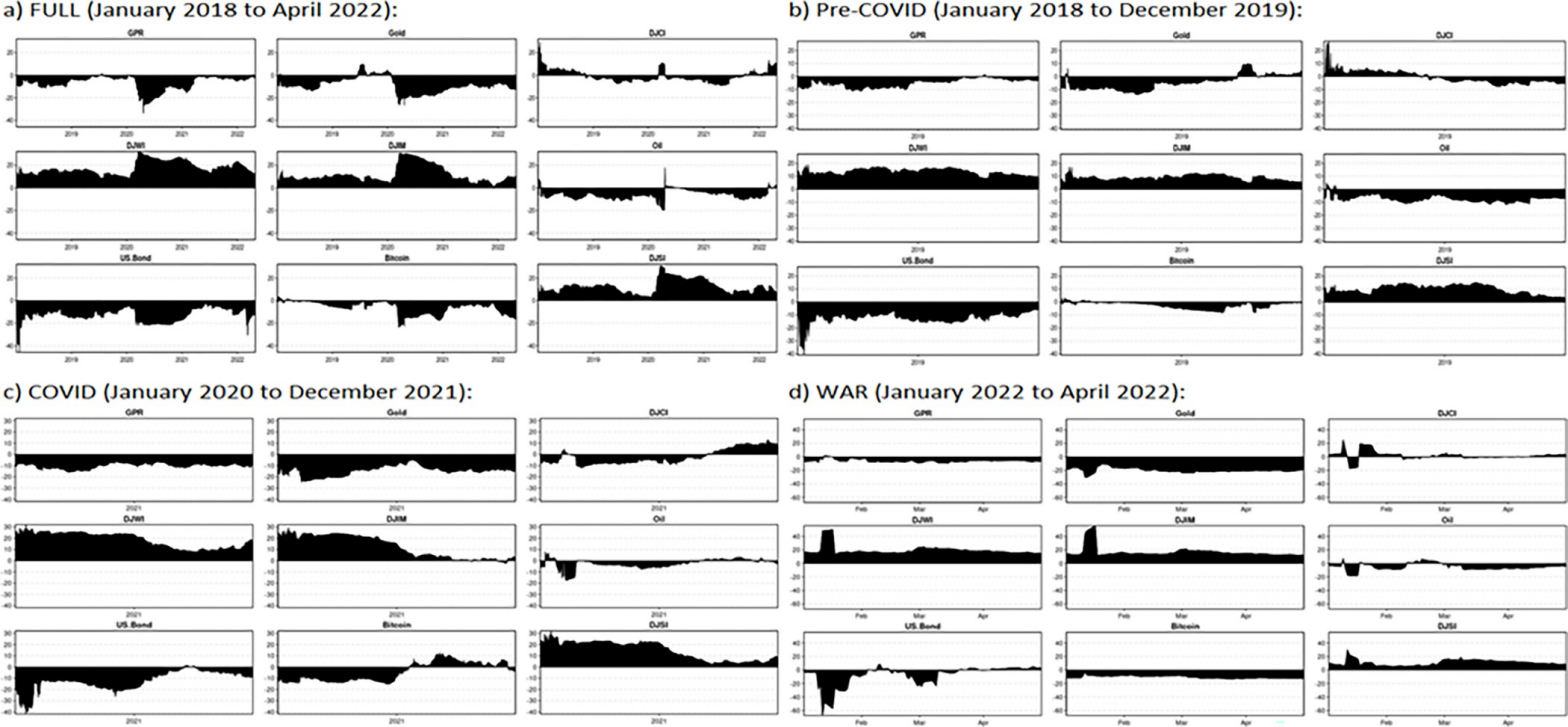
Net total directional connectedness. a) FULL (January 2018 to April 2022): b) Pre-COVID (January 2018 to December 2019) c) COVID (January 2020 to December 2021): d) WAR (January 2022 to April 2022): Note: The X-axis denotes the time line for four different sample periods.

The direction of the net transmittor of volatility to all the other variables will be towards the positive side whereas the net recepient of volatility from all the other variables will be towards the negative side as per the net directional connectedness measure plot. **Fig 4A.** shows that DJWI, DJIM, and DJSI are positive and hence net transmittors of volatility through out the sample period while GPR, US.Bond, and Bitcoin are observed to be net recipients of volatility during the same period. However, Oil which remained to be a net recipient through out the sample period became a net transmittor during early 2020. **Fig 4B.** shows that DJWI, DJIM, and DJSI are positive and hence net transmittors of volatility; whereas DJCI shifted its position from net transmittor to net recipient during the end of year 2018. Further GPR, Bitcoin, US. Bond, and Oil remained to be net recipients of volatility throughout the Pre-COVID sample period. Fig 4C. shows that DJWI, DJIM, and DJSI are net transmittors of volatility and GPR, Gold, and US.Bond are net recipients of volatility through out the COVID-19 sample period. However, we find fluctuations in the movements of volatility net total directional connectedness in DJCI, Oil, and Bitcoin during COVID-19 period. Fig 4D. shows that there are fluctuations in net volatility total directional connectedness in commodity index, oil, and US.Bonds during Russian-Ukraine war period.

**Fig 5:**
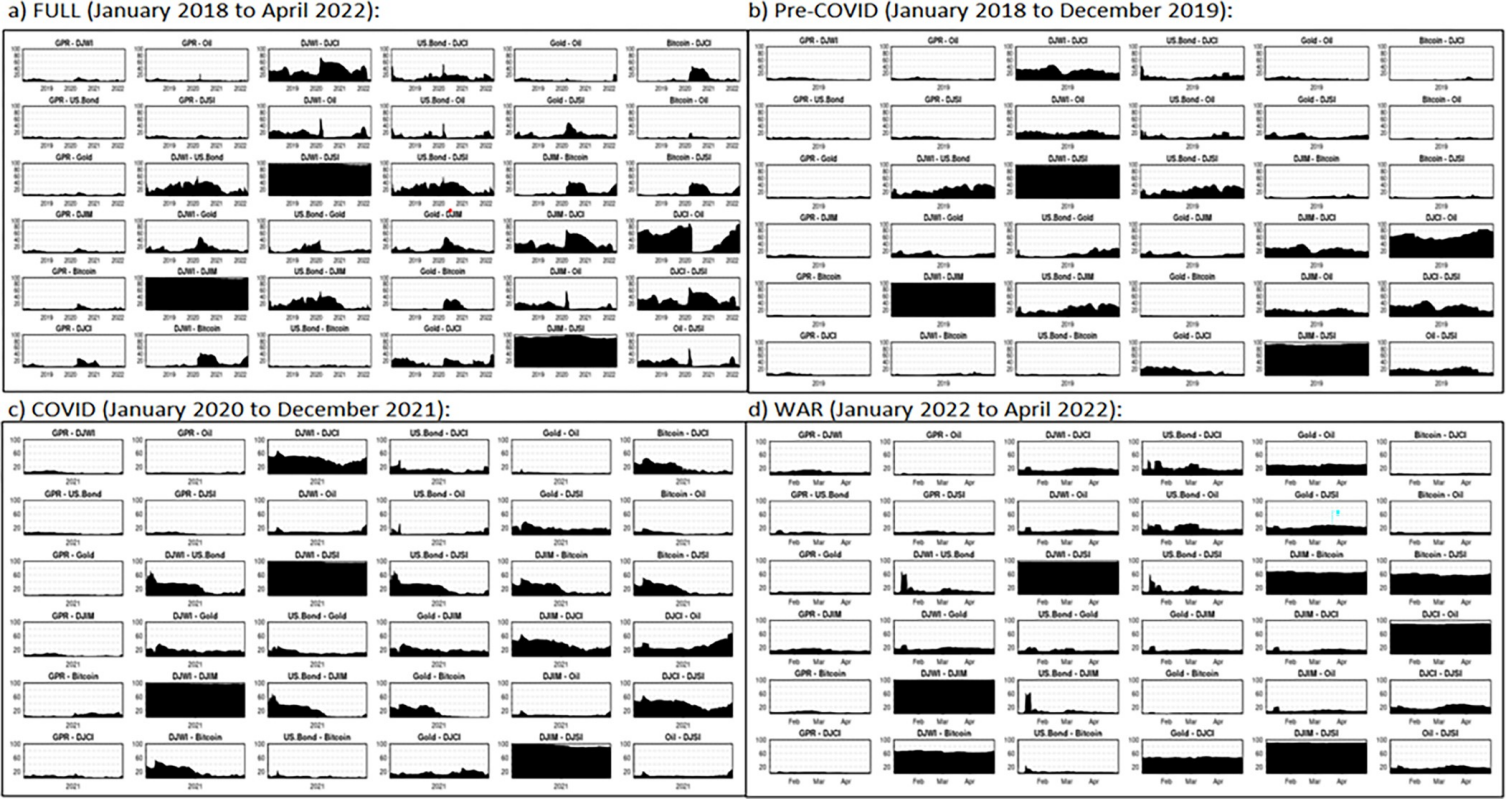
Dynamic pairwise directional connectedness. a) FULL (January 2018 to April 2022): b) Pre-COVID (January 2018 to December 2019): c) COVID (January 2020 to December 2021): d) WAR (January 2022 to April 2022): Note: The X-axis denote the time line for four different sample periods.

**Fig 5A.** shows that the pairwise directional connectedness between stocks (DJWI-DJIM, DJWI-DJSI, DJWI-DJCI, DJIM-DJSI, DJCI-DJSI) is positive and hence we can say that the bilateral relationship between conventional, Islamic, sustainable, and commodity indices is strong throughout the different subsample periods. We found that the bilateral relationship between Gold-Bitcoin, Bitcoin-DJCI, Bitcoin-Oil, Bitcoin-DJSI, US. Bond-Bitcoin was weak during pre-COVID period (**Fig 5B**). The bilateral relationship between Bitcoin- DJCI, and Gold-Bitcoin is positive and strong during COVID-19 period (**Fig 5C**) compared to Russian-Ukraine war period. Further, the bilateral relationship between GPR and other financial assets; Gold-Oil, Gold-DJSI, Gold-DJCI, DJIM-Bitcoin, Bitcoin-DJSI, and DJCI-Oil is observed to be positive and strong during Russian-Ukraine war period (**Fig 5D**) compared to COVID-19 and pre-COVID sample periods.

**Fig 6:**
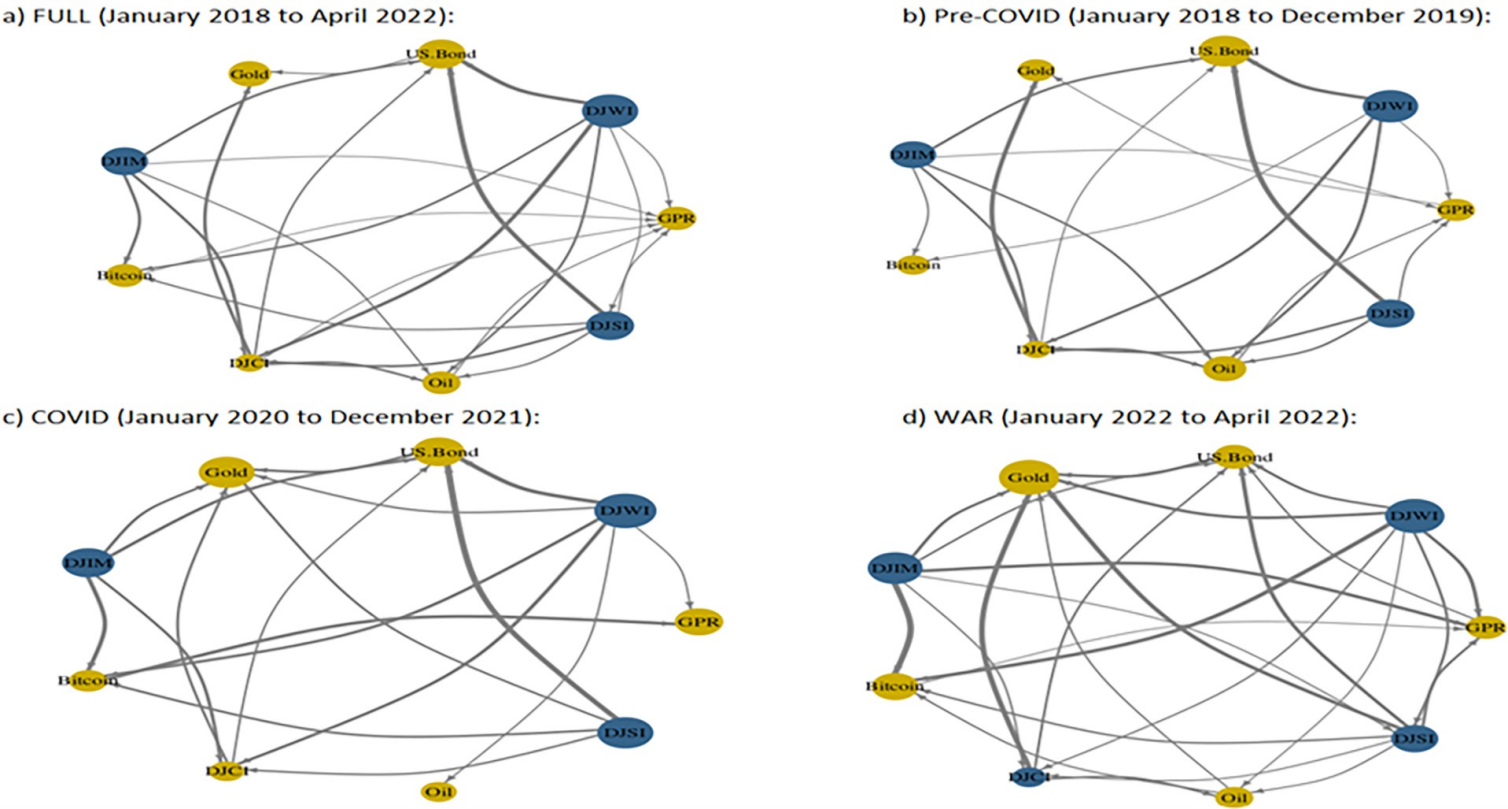
Network plot. a) FULL (January 2018 to April 2022): c) COVID (January 2020 to December 2021): b) Pre-COVID (January 2018 to December 2019): d) WAR (January 2022 to April 2022): **Note:** The net transmittors of volatility are displayed in blue color and the net recipients are shown in yellow color.

**Fig 6** displays the network plot of volatility connectedness. The net transmittors of volatility are displayed in blue color and the net recipients are shown in yellow color. The node’s size shows the magnitude of the contribution of each variable to system connectedness. The thicker lines exhibit greater extent of volatility spillover compared to the thinner lines. The conventional stock (DJWI), Islamic stock (DJIM), and sustainable stock (DJSI) indices are observed to be net transmittors of volatility, whereas Gold, US.Bond, GPR, Oil, and Bitcoin remain to be net recipients of volatility through out the subsample periods. We observe that commodity index (DJCI) shifted from net recipient of volatility to net transmittor during Russian-Ukraine war period. Overall, conventional stock DJWI remained to be strong volatility transmittor and Gold is observed to be strong volatility recipient which also demonstrates the safe haven characteristic of Gold during turbulent times in the financial markets.

## 6. Conclusion

This paper investigates the dynamic connectedness of Geo-Political Risk index, Stocks, Bond, Bitcoin, Gold and Oil for the period from January 2018 to April 2022. Our methodology is inspired by the works of Diebold and Ylmaz, 2012; Gabauer, 2021; Chatziantoniou & Gabauer, 2021; to perform dynamic connectedness that employs DCC-GARCH framework.

We investigate the connectivity during Pre-COVID, COVID, and Russian-Ukraine war sub sample periods. We find that the conventional, Islamic, and sustainable stock indices are net volatility transmittors during COVID-19 and Russian-Ukraine war periods; whereas Gold, US. Bond, GPR, Oil, and Bitcoin are found to be net volatility recipients through out the sample periods. We observe that commodity index (DJCI) shifted from net recipient of volatility to net transmittor during Russian-Ukraine war period. Further, we find that bilateral intercorrelations are strong within stock indices (DJWI, DJIM, and DJSI) and weak among other financial assets.

Our study has beneficial implications for policymakers, regulators, investors, and financial market constituents to redevelop their existing strategies to avoid financial losses. Risk management and policies can be achieved through the control and management of connectedness index. Investors, portfolio managers, and policymakers can develop effective investment strategies and hedges against GPR, as well as conduct risk management. Our discovery of a stronger long-term impact on volatility dynamics suggests that risk transmission from such uncertainty should be taken into account when making long-term asset allocation decisions. Quantifying myopic and intertemporal asset allocation decisions in the face of uncertainty could be the focus of future research.
